# Resuscitation of a Young Girl Apparently Dead from Drowning

**Published:** 1879-08-20

**Authors:** E. L. Godfrey

**Affiliations:** Camden, New Jersey


### *REPORT OF THE RESUSCITATION OF A YOUNG GIRL APPARENTLY DEAD FROM DROWNING.*

BY E. L. GODFREY, M. D., CAMDEN, NEW JERSEY.

The patient was a young girl, twelve years of age, of vigorous constitution, just rescued, in an insensible condition, from the water. The exact time in which she was in the water could not be accurately ascertained, but when rescued she was cold, pale, pulseless, with muscular system completely relaxed, with no signs of breathing, animation to all appearances being completely suspended.

After placing the patient upon her back, clearing the mouth and throat of frothy mucus, drawing forward the tongue, and loosening everything about the neck, both arms were raised as far as possible above the head and quickly brought down to the sides, while the chest was forcibly compressed and the patient turned quickly over, face downwards, so as to admit of the escape of fluid. These manipulations were twice quickly done, and had the desired effect of forcing considerable water and mucus froth from the air-passages. After the escape of the water, the patient was placed upon her back, the tongue again drawn forward, and the movement of the arms, according to the method of Dr. Sylvester, with manual compression of the chest, was repeated, first slowly, then gradually increased to about twenty times per minute, with all possible regularity and gentleness. Efforts to promote warmth of body and activity of circulation were not unduly made until signs of returning animation were observed, which were gasping, convulsive tremors of the muscles of the face, and vomiting. After these, the limbs were vigorously rubbed, chiefly towards the heart; dry warmth applied to the groins and axillæ; sinapisms to the extremities, along the spine, and over the precordial region; ammonia passed under the nostrils, and flagellation, so highly and justly extolled by Professor Gross, vigorously applied by my assistants. Artificial respiration was continued all this time. After vomiting occurred, efforts at breathing became more marked, from greater play allowed the diaphragm. Then, after two hours of hard work, I was rewarded by the appearance of the unmistakable signs of returning animation. Stimulants were administered, *pro re nata*, as soon as patient could swallow, and when breathing and consciousness became well restored, patient was placed in a warm bed. Every indication to over-action was promptly met by the usual remedies, and the next morning the patient was convalescent.

*Remarks.*—In this case I believe manual compression of the chest, which forced the carbonic acid from the lungs, and mucus froth and water from the air-passages, and thus allowed the free ingress of air, contributed very materially towards the establishment of respiration.

The manipulation of the chest was easy of execution, owing to its size. Manual compression of the chest was more particularly brought to the notice of the profession by Dr. Benjamin Howard, of New York, in an article read before the American Medical Association in 1871, and is certainly a very material modification of the method of Dr. Sylvester, of London. The extensive inquiries instituted by the Royal National Life-Boat Institution of England, among medical men and medical bodies, have been the means of establishing a number of principles and directions to be carried out in effecting the restoration of the apparently dead from drowning. These principles are more especially founded (since the abandonment of Marshall Hall's) on those devised by Dr. Sylvester. Sylvester's method is too well known to need mention, but its active employment, if persistently followed, will often give the most flattering results, as is shown in the case reported, and also in a case reported by Mr. Douglas, in which no evidence of respiration was perceived until after the manipulations had been uninterruptedly performed for eight and a half hours. The period at which people die during submersion varies, of course, but recovery is said to be doubtful after a submersion of two minutes. Animation, however, is not completely suspended at once, unless the person is overcome by fright, injury, or coldness of the air and water. Even the Navarino sponge-divers are said not to be able to sustain submersion longer than twenty-six seconds, while the pearl-divers of Ceylon, according to Mr. Marshall, can seldom remain under water longer than two-thirds of that time. Syncope during submersion is favorable to recovery, as the system in that state is not in as urgent need of oxygen as in its active state. This is well shown in the case reported by Dr. Wooley, in which a girl, while in a state of syncope, fell into the water, and was restored to life after having been submerged six minutes.

Experimentation has shown that drowning is more speedily fatal to life than ordinary suffocation; that the chances of recovery are lessened from the effects produced on the lungs by water; that the lungs become passively congested; that as unconsciousness approaches, during submersion, water enters the air-passages and air-cells of the lungs, and that, from the congestion of the lungs and water in the air-cells, there results a bloody, frothy mucus, which not only fills up the air-cells and blocks up the smaller bronchial tubes, but penetrates the intimate structure of the lungs, rendering them œdematous, and thus physically unfit to receive or expel air by respiration. Thus, in cases of apparent death from drowning, the chances of recovery depend less upon the action of the heart than upon the quantity of frothy mucus present in the air-passages and its penetration into the intimate structure of the lungs.—*Medical Times.*

